# Effects of trastuzumab and trastuzumab emtansine on corrected QT interval and left ventricular ejection fraction in patients with metastatic (HER2+) breast cancer

**DOI:** 10.1186/s43044-023-00331-y

**Published:** 2023-02-13

**Authors:** Rea Levicki, Martina Lovrić Benčić, Irena Ivanac Vranešić, Lada Bradić, Marta Begovac, Juraj Jug, Natalija Dedić Plavetić

**Affiliations:** 1 Department of Cardiology, Požega General Hospital, Osjecka Street 107, 34 000 Požega, Croatia; 2 Department of Cardiovascular Diseases, University Hospital Centre Zagreb, University of Zagreb School of Medicine, 10 000 Zagreb, Croatia; 3 Department of Cardiovascular Diseases, University Hospital Centre Zagreb, 10 000 Zagreb, Croatia; 4 University of Zagreb School of Medicine, 10 000 Zagreb, Croatia; 5 Department of Medical Oncology, University Hospital Centre Zagreb, University of Zagreb School of Medicine, 10 000 Zagreb, Croatia

**Keywords:** Trastuzumab, Cardiotoxicity, Breast cancer

## Abstract

**Background:**

Trastuzumab and trastuzumab emtansine are specific antibody and antibody–drug conjugates used in the treatment of human epidermal growth factor receptor 2 (HER2) positive metastatic breast cancer. The aim of this study was to test their effect on the QTc interval duration and left ventricular ejection fraction (LVEF) in our patients, two parameters used in evaluation of cardiotoxicity. From May 2015 to October 2017, 26 patients with preserved LVEF were included in the study. All of them were previously treated with standard paclitaxel and cisplatin-based chemotherapy regimens. Electrocardiogram (ECG) was recorded just before each trastuzumab dose application and six months after the last dose. Echocardiography with LVEF measurement was performed several days before the application of the initial dose, and six months after the last cycle. Later, 24 patients with metastatic disease received additional treatment with trastuzumab emtansine after six months and the same ECG and echocardiography protocol was performed again. Due to reduction in LVEF, two patients were discontinued from additional treatment.

**Results:**

A statistically significant QTc prolongation was found after each drug dose application, with an increase in mean QTc duration with every successive application, reaching the peak QTc values just before the fifth cycle of treatment. The QTc interval returned to its initial value six months after the last cycle (*p* < 0.001). These results were similar for both drugs. Mean LVEF before both treatment protocols was significantly higher compared to LVEF value after the treatment. LVEF before trastuzumab emtansine treatment was non-significantly higher than LVEF after trastuzumab treatment.

**Conclusion:**

Trastuzumab and trastuzumab emtansine cardiotoxicity manifested as a significant and progressive QTc prolongation after successive drug applications, reaching the peak value just before the fifth cycle of both drugs. Both medications also caused statistically significant but asymptomatic LVEF reduction. Complete reversibility of cardiotoxic effects of both drugs was confirmed by QTc interval and LVEF normalisation after the treatment discontinuation.

**Supplementary Information:**

The online version contains supplementary material available at 10.1186/s43044-023-00331-y.

## Background

Breast cancer is the most frequently diagnosed cancer in women worldwide, with the highest mortality rate among malignant diseases [1]. Twenty percent of breast cancers overexpress human epidermal growth factor receptor 2 (HER2), a transmembrane glycoprotein that serves as epidermal growth factor receptor (EGFR) with tyrosine-kinase activity. Therapies targeting HER2 are paramount in metastatic breast cancer management [2]. Improved survival in patients with metastatic HER2-positive breast cancer warrants their use in first-line and subsequent line treatment [3]. There are four HER2-directed agents: trastuzumab—a monoclonal antibody that binds the extracellular domain of HER2, ado-trastuzumab emtansine (T-DM1)—an antibody–drug conjugate composed of trastuzumab, a thioether linker, and a derivative of antimitotic agent maytansine, and also lapatinib and pertuzumab [4]. Randomized trials suggest non-inferiority of trastuzumab monotherapy in comparison to the combination of trastuzumab and chemotherapy [5–7]. Patients who relapse within six months of completing adjuvant trastuzumab therapy are eligible for ado-trastuzumab emtansine treatment [8]. Trastuzumab is associated with cardiotoxic risk, mostly manifested as an asymptomatic decrease in left ventricular ejection fraction (LVEF), and less commonly as symptomatic heart failure [9–11]. Two types of chemotherapy-related cardiac dysfunction have been described. Type I, in association with anthracycline use, manifests as partial myocyte destruction and clinical heart failure, while type II, associated with trastuzumab use, shows myocardial hibernation, probably not associated with myocyte death or clinical heart failure, and is therefore reversible [12]. Incidence of trastuzumab-related cardiotoxicity varies according to other comorbidities [13]. Important risk factors that contribute to trastuzumab-related cardiotoxicity are: age above 50 years, previous anthracycline use, and obesity [14–18]. Due to the high incidence rate of trastuzumab-related cardiotoxicity in patients with metastatic breast cancer, it is recommended to assess cardiac function (LVEF) before treatment, and in case of new onset heart failure symptoms [9, 11, 14, 19]. It is also recommended to obtain and analyze the electrocardiogram (ECG) before every trastuzumab and trastuzumab emtansine treatment. The long-term effect of trastuzumab on QT dispersion (QTd) was investigated in a pilot study showing significantly higher QTd in patients treated with trastuzumab after anthracycline-based regimen compared to patients treated with anthracycline-based regimen only (0.064 ± 0.023 s vs. 0.051 ± 0.016, *p* = 0.034) [20]. Also, mean increases in QTd and QTcd are significantly different in patients treated with paclitaxel-trastuzumab combination (0.021 ± 0.011 and 0.022 ± 0.014 s, respectively) compared to those treated with anthracycline-based regimen (0.005 ± 0.003 and 0.006 ± 0.008 s, respectively) in the same group (*p* = 0.0246) (20). Trastuzumab in long-term management can significantly prolong the QT interval, while a significant QT interval prolongation was not previously documented in trastuzumab emtansine treatment protocols [20, 21]. The aim of this study was to determine the effects of trastuzumab and trastuzumab emtansine on the QTc interval and LVEF.

## Methods

In this prospective cohort study, we investigated 65 women with metastatic (HER2+) breast cancer treated in our Department of Medical Oncology, University Hospital Centre Zagreb, Croatia. All patients underwent treatment with the standard chemotherapy regimen which included paclitaxel and cisplatin. Thirty-nine women were excluded from the second-line treatment due to poor clinical condition or fatal outcome. We gathered data from 26 patients who continued treatment with the specific antibody drug. The average age of our subjects was 57.96 years (± 7.48 years). Eligible patients were treated with six cycles of trastuzumab, as a first-line antibody drug treatment, followed by six cycles of trastuzumab emtansine as a second-line antibody drug treatment protocol, which is the standard in our institution. Both drugs were administered subcutaneously. Only 24 patients with preserved LVEF received the second-line treatment with the antibody drug, trastuzumab emtansine after six months since two patients had LVEF reduction, In addition, one patient developed left bundle branch block, and the other one reached the age limit (70 years). The electrocardiogram was obtained and analyzed before each drug application, and six months after the last application of trastuzumab and trastuzumab emtansine (mean heart rate 72 ± 12/min). Corrected QT values were calculated using Fridericia’s formula (QTc = QT interval/^3^√(60/heart rate). At the time of the ECG recordings, the patients were not on any antiarrhythmics, beta blockers, psychoactive drugs or antibiotics that could influence QT interval duration. All patients had normal serum sodium and potassium levels. Three women had diabetes and were treated with insulin, four women had arterial hypertension and were treated with the ACE inhibitor Ramipril and the Calcium channel blocker Amlodipine. The other patients had no comorbidities. Before the first application of trastuzumab and trastuzumab emtansine, as well as six months after the last cycle, an echocardiogram was performed in order to assess LVEF using the biplane Simpson’s method. Data was presented as frequencies, mean with standard deviation (SD) and median with 5th and 95th percentiles, as appropriate. For statistical analysis we used Student's *t*-test of paired samples to compare QTc intervals before each cycle and after the last application of both drugs. The Bayesian Pearson correlation test was used to examine the correlation between QTc intervals and LVEF. A statistically significant *p*-value of < 0.05 was used.

## Results

ECG tracing was obtained before each drug application and six months after the last cycle in 26 patients treated with trastuzumab, and 24 patients treated with trastuzumab emtansine. The results of ECG analysis are shown below (Tables 1, 2, 3 and 4).

**Table 1:** PQ interval duration before each trastuzumab/trastuzumab emtansine cycle and six months after the last cycle

Number of cycle	Trastuzumab	Trastuzumab emtansine
	PQ interval mean values ± SD (ms)	PQ interval(ms)
PQ1	161 ± 22.62	160.2 ± 23.56
PQ2	160.6 ± 22.61	160.5 ± 22.48
PQ3	159.7 ± 21.48	159.8 ± 22.3
PQ4	160.6 ± 22.99	161.4 ± 24.71
PQ5	160.2 ± 22.46	160.6 ± 23.14
PQ6	159.7 ± 22.6	159.9 ± 23.69
PQ7 (after 6 months)	159.8 ± 21.53	159.8 ± 22.42

There were no significant changes in the PQ interval duration, neither in relation to subsequent trastuzumab applications nor 6 six months after the last cycle. Comparable to trastuzumab, no significant changes of the PQ interval were found neither in relation to trastuzumab emtansine cycles, nor six months after the last application of the drug (Table 1)

**Table 2:** QRS interval values before each trastuzumab/trastuzumab emtansine application and 6 months after the last cycle

Number of cycle	TRASTUZUMAB	TRASTUZUMAB EMTANSINE
	QRS interval mean values (mean ± SD) (ms)	QRS interval values (ms)
QRS1	100.8 ± 11.16	99.4 ± 7.07
QRS2	100.8 ± 10.36	99.4 ± 6.75
QRS3	101.2 ± 10.23	99.8 ± 6.45
QRS4	101.6 ± 10.25	100.3 ± 6.58
QRS5	101.7 ± 10.15	100.4 ± 6.69
QRS6	101.6 ± 10.21	100.4 ± 6.71
QRS7 (after 6 months)	101.4 ± 10.30	100.1 ± 6.78

QRS interval values showed no statistically significant difference between trastuzumab/trastuzumab emtansine cycle. The same was found six months after the last application

**Table 3:** QTc interval comparison between different trastuzumab cycles using *t*-test of paired samples

Trastuzumab	Results of QTc interval comparison using *t*-test of paired samples				
QTc1 versus QTc2	QTc1 449.4 (23.26)	QTc2 452.4 (20.73)	*t* = − 3.25	df = 25	*p* = 0.003
QTc1 versus QTc3	QTc1 449.4 (23.26)	QTc3 455.1 (21.08)	*t* = − 6.19	df = 25	*p* < 0.001
QTc1 versus QTc4	QTc1 449.4 (23.26)	QTc4 472.1 (32.08)	*t* = − 6.19	df = 25	*p* < 0.001
QTc1 versus QTc5	QTc1 449.4 (23.26)	QTc5 473 (27.79)	*t* = − 7.96	df = 25	*p* < 0.001
QTc1 versus QTc6	QTc1 449.4 (23.26)	QTc6 471.3 (28.58)	*t* = − 6.5	df = 25	*p* < 0.001
QTc1 versus QTc7	QTc1 449.4 (23.26)	QTc7 453 (20.42)	*t* = − 3.57	df = 25	*p* = 0.001
QTc2 versus QTc3	QTc2 452.4 (20.73)	QTc3 455.1 (21.08)	*t* = − 3.87	df = 25	*p* < 0.001
QTc2 versus QTc4	QTc2 452.4 (20.73)	QTc4 472.1 (32.08)	*t* = − 5.02	df = 25	*p* < 0.001
QTc2 versus QTc5	QTc2 452.4 (20.73)	QTc5 473 (27.79)	*t* = − 6.67	df = 25	*p* < 0.001
QTc2 versus QTc6	QTc2 452.4 (20.73)	QTc6 471.3 (28.58)	*t* = − 5.42	df = 25	*p* < 0.001
QTc2 versus QTc7	QTc2 452.4 (20.73)	QTc7 453 (20.42)	*t* = − 0.67	df = 25	*p* = 0.507
QTc3 versus QTc4	QTc3 455.1 (21.08)	QTc4 472.1 (32.08)	*t* = − 4.29	df = 25	*p* < 0.001
QTc3 versus QTc5	QTc3 455.1 (21.08)	QTc5 473 (27.79)	*t* = − 5.84	df = 25	*p* < 0.001
QTc3 versus QTc6	QTc3 455.1 (21.08)	QTc6 471.3 (28.58)	*t* = − 4.67	df = 25	*p* < 0.001
QTc3 versus QTc7	QTc3 455.1 (21.08)	QTc7 453 (20.42)	*t* = 1.91	df = 25	*p* = 0.067
QTc4 versus QTc5	QTc4 472.1 (32.08)	QTc5 473 (27.79)	*t* = − 0.78	df = 25	*p* = 0.443
QTc4 versus QTc6	QTc4 472.1 (32.08)	QTc6 471.3 (28.58)	*t* = 0.59	df = 25	*p* = 0.556
QTc4 versus QTc7	QTc4 472.1 (32.08)	QTc7 453 (20.42)	*t* = 4.71	df = 25	*p* = 0.556
QTc5 versus QTc6	QTc5 473 (27.79)	QTc6 471.3 (28.58)	*t* = 1.96	df = 25	*p* = 0.061
QTc5 versus QTc7	QTc5 473 (27.79)	QTc7 453 (20.42)	*t* = 6.17	df = 25	*p* < 0.001
QTc6 versus QTc7	QTc6 471.3 (28.58)	QTc7 453 (20.42)	*t* = 5.01	df = 25	*p* < 0.001

Student's *t*-test of paired samples was also used to compare QTc interval values obtained before each trastuzumab emtansine cycle and after the last application of the drug. A statistically significant *p* value of < 0.05 was used

**Table 4:** QT interval comparison between trastuzumab emtansine cycles using *t*-test of paired samples

Trastuzumab emtansine	Results of QTc interval comparison using *t*-test of paired samples				
QTc1 versus QTc2	QTc1 448.6 (18.52)	QTc2 453.6 (18.44)	*t* = − 7.28	df = 23	*p* < 0.001
QTc1 versus QTc3	QTc1 448.6 (18.52)	QTc3 458.3 (19.19)	*t* = − 9.34	df = 23	*p* < 0.001
QTc1 versus QTc4	QTc1 448.6 (18.52)	QTc4 470.1 (26.72)	*t* = − 6.12	df = 23	*p* < 0.001
QTc1 versus QTc5	QTc1 448.6 (18.52)	QTc5 474.5 (28.95)	*t* = − 6.78	df = 23	*p* < 0.001
QTc1 versus QTc6	QTc1 448.6 (18.52)	QTc6 471.9 (25.56)	*t* = − 6.69	df = 23	*p* < 0.001
QTc1 versus QTc7	QTc1 448.6 (18.52)	QTc7 454.4 (16.91)	*t* = − 4.54	df = 23	*p* < 0.001
QTc2 versus QTc3	QTc2 453.6 (18.44)	QTc3 458.3 (19.19)	*t* = − 5.43	df = 23	*p* < 0.001
QTc2 versus QTc4	QTc2 453.6 (18.44)	QTc4 470.1 (26.72)	*t* = − 5.43	df = 23	*p* < 0.001
QTc2 versus QTc5	QTc2 453.6 (18.44)	QTc5 474.5 (28.95)	*t* = − 5.78	df = 23	*p* < 0.001
QTc2 versus QTc6	QTc2 453.6 (18.44)	QTc6 471.9 (25.56)	*t* = − 5.54	df = 23	*p* < 0.001
QTc2 versus QTc7	QTc2 453.6 (18.44)	QTc7 454.4 (16.91)	*t* = − 0.58	df = 23	*p* = 0.56
QTc3 versus QTc4	QTc3 458.3 (19.19)	QTc4 470.1 (26.72)	*t* = − 3.7	df = 23	*p* = 0.001
QTc3 versus QTc5	QTc3 458.3 (19.19)	QTc5 474.5 (28.95)	*t* = − 4.62	df = 23	*p* < 0.001
QTc3 versus QTc6	QTc3 458.3 (19.19)	QTc6 471.9 (25.56)	*t* = − 4.29	df = 23	*p* < 0.001
QTc3 versus QTc7	QTc3 458.3 (19.19)	QTc7 454.4 (16.91)	*t* = 3.13	df = 23	*p* = 0.005
QTc4 versus QTc5	QTc4 470.1 (26.72)	QTc5 474.5 (28.95)	*t* = − 4.63	df = 23	*p* < 0.001
QTc4 versus QTc6	QTc4 470.1 (26.72)	QTc6 471.9 (25.56)	*t* = − 1.59	df = 23	*p* = 0.124
QTc4 versus QTc7	QTc4 470.1 (26.72)	QTc7 454.4 (16.91)	*t* = 4.62	df = 23	*p* < 0.001
QTc5 versus QTc6	QTc5 474.5 (28.95)	QTc6 471.9 (25.56)	*t* = 2.09	df = 23	*p* = 0.047
QTc5 versus QTc7	QTc5 474.5 (28.95)	QTc7 454.4 (16.91)	*t* = 5.33	df = 23	*p* < 0.001
QTc6 versus QTc7	QTc6 471.9 (25.56)	QTc7 454.4 (16.91)	*t* = 5.27	df = 23	*p* < 0.001

Student’s *t*-test of paired samples was also used to compare QTc interval values obtained before each trastuzumab emtansine cycle and after the last application of the drug. A statistically significant *p* value of < 0.05 was used

QTc interval duration showed progressive prolongation with each successive trastuzumab application, with insignificant shortening of the QTc6 interval, measured before the 6th trastuzumab application, while QTc values six months after the last application (QTc7) returned back to the initial values. QTc intervals also showed progressive prolongation with each successive trastuzumab emtansine application, with the exception of QTc6 interval shortening (obtained before the 6th trastuzumab emtansine application), while QTc values six months after the last application returned approximately to the initial values.

Student's *t*-test of paired samples was used to compare QTc interval values before each trastuzumab cycle and after the completion of therapy. A significant QT interval prolongation with every trastuzumab application was found, with a mean QTc1 duration of 449.4 ± 23.26 ms, QTc2 (before the second cycle) of 452.4 ± 20.73 ms, QTc3 (before the third cycle) 455.1 ms (± 21.08 ms), QTc4 (before the fourth cycle) 472.1 ms (± 32.08 ms), QTc5 (before the fifth cycle) 473 ms (± 27.79 ms) (*p* < 0,001). An insignificant shortening of the QTc6 interval before the sixth trastuzumab application was found, mean 471.3 ms (± 28.58 ms), while the mean QTc value six months after the last cycle (QTc7) returned almost to starting values 453 ms (± 20.42 ms) (*p* < 0,001) (Table 3).

Using the Bayesian Pearson correlation test with positive correlation presumption, we compared QTc1 interval values with LVEF values measured before trastuzumab treatment, and found no positive correlation; results show an insignificant negative correlation (*r* = − 0.125, BF10 = 0.162). In the comparison of QTc7 interval values with EF2 values measured after trastuzumab treatment, there was also no positive correlation found; results show an insignificant negative correlation (*r* = − 0.171, BF10 = 0.143). When comparing QTc1 interval values with EF1 values measured before trastuzumab emtansine treatment, results showed an insignificant negative correlation (*r* = − 0.108, BF10 = 0.286). Also, in comparison of QTc7 interval values with EF2 values measured after trastuzumab emtansine treatment, we found an insignificant negative correlation (*r* = − 0.170, BF10 = 0.341).

## Discussion

Results of ECG analysis in 26 patients treated with trastuzumab and 24 patients who continued with trastuzumab emtansine treatment showed no significant effect of the given treatment on PQ and QRS interval values (Tables 1 and 2), similar to previous findings [22]. In addition, an earlier study on pertuzumab effects on PQ and QRS intervals also found no significant drug effect on the aforementioned intervals [23]. Other published data show discordant results regarding QTc interval prolongation after trastuzumab and trastuzumab emtansine treatment. Several studies found no clinically relevant effect on QTc in relation to trastuzumab treatment [22], while other publications found significant QT and QTc interval prolongation in patients with breast cancer, as a side effect of both acute and long-term treatment with trastuzumab [20, 24]. The risk of trastuzumab-related cardiac dysfunction was found to be higher in patients previously treated with anthracyclines [14–18]. It is important to emphasize that our patients were pretreated with paclitaxel and cisplatin before start of trastuzumab and trastuzumab emtansine. Studies have also shown an association between paclitaxel and ventricular arrhythmias, bradycardia, several degrees of atrioventricular conduction block, bundle branch block (effects mediated by paclitaxel vehicle Cremophor EL) as well as cardiac ischemia. In protocols involving the combination of doxorubicin and paclitaxel, cardiotoxicity was attributed to doxorubicin alone. Cisplatin has been shown to be associated with an increased risk of thrombotic events, deep vein thrombosis and pulmonary embolism, although specific cardiotoxicity is rarely reported [25]. In patients treated with doxorubicin and trastuzumab, results suggest a combined cardiotoxicity effect associated with the cumulative dose of doxorubicin; concurrent application of both drugs is expected to be safe if cumulative dose is limited to 180 mg/m^2^ or less [26–29]. Anthracycline induced-cardiotoxicity varies from 4% to over 36% in patients receiving a dose of 500–550 mg/m^2^. Main cardiotoxic mechanism is mediated by free radical formation, while apoptosis plays a significant role in myocardial cell loss. Cardiotoxicity can be categorized into acute (transient decline in myocardial contractility immediately after the infusion, incidence < 1%), early onset chronic progressive toxicity (within the first year from the completion of treatment, incidence 1.6–2.1%) or late onset chronic progressive toxicity (presenting as dilated cardiomyopathy (CMP) at least one year following the completion of therapy) [25]. The incidence of trastuzumab-related heart failure has been found to be 2–7%, with an increase to 27% with prior treatment with anthracyclines, if trastuzumab is used concurrently with anthracycline plus cyclophosphamide. Trastuzumab toxicity is not dose-dependent and is frequently reversible, in contrast to anthracycline cardiotoxicity [25]. There is currently not enough data on cardiotoxicity pattern of trastuzumab emtansine, not even from a trial including a large number of patients However, more adverse events were associated with trastuzumab emtansine than with trastuzumab alone. [30]

**Fig. 1:**
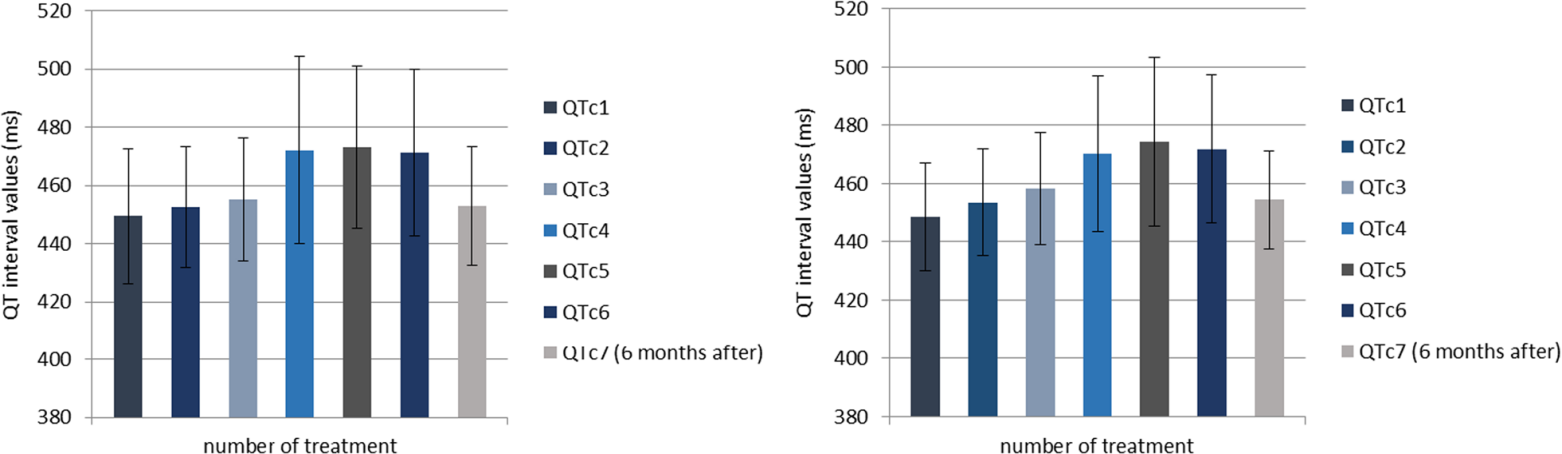
QTc interval values before and 6 months after the last trastuzumab (left)/trastuzumab emtansine cycle (right)

**Fig. 2:**
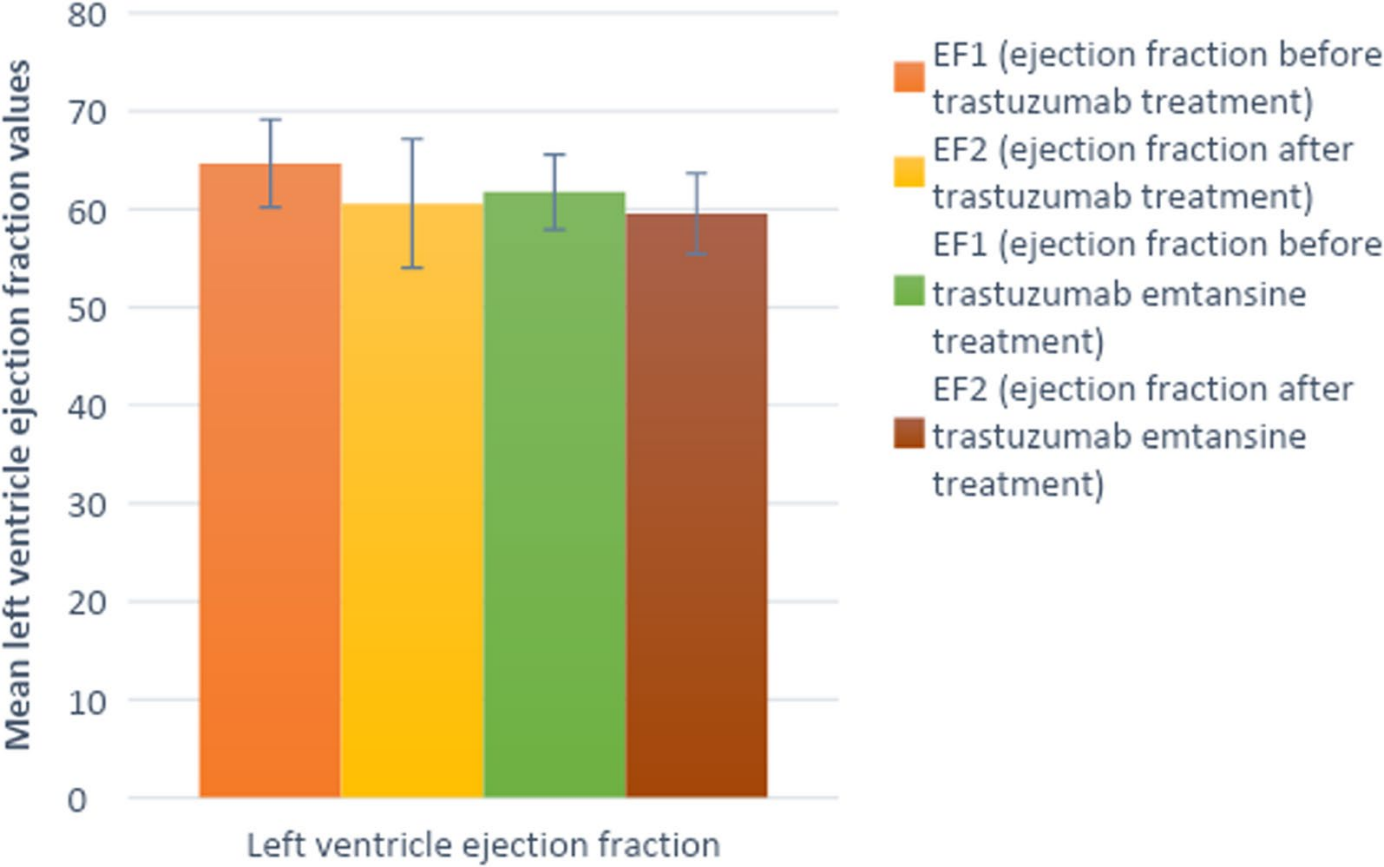
LVEF values measured before and after both antibody drug protocols (*LVEF1 T* Left ventricular ejection fraction before trastuzumab, *LVEF2 T* Left ventricular ejection fraction after trastuzumab, *LVEF1 ET* Left ventricular ejection fraction before trastuzumab emtansine, *LVEF2 ET* Left ventricular ejection fraction after trastuzumab emtansine)

Our study found a significant QTc interval prolongation with every cycle of trastuzuma (Table 3, Fig. 1). An insignificant shortening of the QTc6 interval, before the sixth trastuzumab treatment, was found, while QTc values six months after the last cycle returned to almost starting values (mean QTc7 453 ms ± 20.42 ms), (Table 3, Fig. 1). A significant QTc interval prolongation was found after the very first trastuzumab cycle, and the QTc interval continued to prolong after each new dose, reaching the peak value before the fifth application (mean QTc5 473 ± 27.79 ms), (Table 3, Fig. 1). According to previous research, the mechanism of trastuzumab cardiotoxicity is presumed to be type II toxicity, which is not associated with myocyte death. Therefore, it was expected for QTc7 interval values (six months after the last dose of trastuzumab) to return to the initial values. We confirmed these findings from the previous studies regarding reversibility of trastuzumab-related cardiotoxic effects [12]. The results of one multicenter study on the safety and pharmacokinetic characteristics of trastuzumab emtansine in female patients with HER2-positive metastatic breast cancer showed a clinically irrelevant effect on QTc interval [21]. Our study, on the other hand, showed a progressive prolongation of the QTc interval duration with each trastuzumab emtansine cycle reaching the highest values before the fifth cycle with a mean of 474.5 ms (± 28.95 ms). This was followed by shortening of the QTc6 interval before the sixth trastuzumab emtansine application to a mean of 471.9 ms (± 25.56 ms). Finally the QTc values six months after the last application returned approximately to the basal values of QTc7 with a mean of 454.4 ms (± 16.91 ms) (Table 4, Fig. 1). These results are similar to the previously mentioned data for trastuzumab alone (Fig. 1). The QTc interval after trastuzumab emtansine administration significantly increased after the first drug treatment and then continued to do so with each successive drug cycle, reaching the peak value before the fifth cycle and then significantly decreasing before the sixth cycle, which is in contrast to the same cycle of trastuzumab alone. Six months posttreatment, the QTc interval values also returned to approximately same starting values (Fig. 1). Based on these results, we presume that trastuzumab emtansine has the same type II cardiotoxicity effect as trastuzumab, without myocyte death in pathogenesis [12]. Many studies have shown an asymptomatic decrease in LVEF as the most common trastuzumab-related cardiotoxicity effect, less often presenting as clinical heart failure [9–11]. Our results show statistically significant LVEF decrease after the trastuzumab treatment, in comparison to initial pretreatment values (EF1 mean 64.69 ± 4.84 vs EF2 mean 60.58 ± 6.58, *t* = 4.96, df = 25, *p* < 0.001), but this reduction was also asymptomatic in our patient cohort (Tables 5 and 6, Fig. 2). A similar cardiotoxic effect was found after trastuzumab emtansine treatment, with a statistically significantly lower LVEF following the protocol, in comparison to initial values (EF1 mean 61.75, SD 3.85, EF2 mean 59.54, SD 4.12, *t* = 7.65, df = 23, *p* < 0.001) (Tables 5 and 6, Fig. 2 but with no clinical repercussions). In our study, we have shown statistically significant effect on LVEF reduction related to both drugs (Fig. 2). One randomized trial of first-line trastuzumab emtansine versus trastuzumab and docetaxel reported an asymptomatic decline in LVEF [31]. Our results also demonstrate a significantly higher LVEF before trastuzumab than before trastuzumab emtansine treatment, and an insignificantly higher LVEF after trastuzumab than after trastuzumab emtansine treatment, possibly reflecting a cumulative cardiotoxic effect of these drugs. As LVEF values before trastuzumab emtansine were insignificantly higher than LVEF after trastuzumab treatment (LVEF2 T mean 60.58, SD 6.58, LVEF1 TE mean 61.75, SD 3.85, *p* = 0.29), we could speculate some degree of LVEF recovery after the trastuzumab treatment (Table 6, Fig. 2). In addition, many studies have shown trastuzumab-related toxicity to be mostly reversible [10, 17, 32–34]. As expected, our results did not show any positive correlation between QT interval and LVEF, before or after both drug protocols. An insignificant negative correlation was found instead. Finally, it is important to notice that most of our patients with metastatic breast cancer, considering their clinical condition, also received other symptomatic therapy, such as antidepressants (amitriptyline, desipramine, imipramine, fluoxetin), antipsychotics (sertindole, ziprasidone, risperidone, citalopram) or antibiotics (quinolone: levofloxacin, moxifloxacin), macrolides (erythromycin, clarithromycin), antimalarials (quinine), antiprotozoal (pentamidine) or antifungal (azole group) medications for acute infections. Some of these medications might also cause QTc interval prolongation which may increase the risk of sudden cardiac death (SCD) due to polymorphic tachycardia, also known as torsades de pointes (TdP) [35]. Ventricular repolarization prolongation often leads to oscillations of the membrane potential called early after-depolarization (EAD), which, in case it reaches a critical threshold in a large myocardial area, can promote ectopic activity [35, 36]. Those ectopic beats are usually followed by a long pause, with a subsequent sinus beat showing marked QTc prolongation. Timing of the ventricular premature contraction (VPC) that occurs on the T-wave of a preceding QRS complex, can trigger an episode of TdP. This pattern of onset of a short-long-short cycle is typical for drug-induced TdP, also known as pause-dependant TdP [35, 37]. This is a pattern where TdP often follows a sudden adrenergic surge, such as exercise or arousal. It is usually not sustained and terminates spontaneously. However, successive events can degenerate into ventricular fibrillation and result in SCD [38].

**Table 5:** LVEF values measured before and after both antibody drug protocols

LVEF	Mean ± SD (%)
LVEF1 T	64.69 (SD 4.48)
LVEF2 T	60.58 (SD 6.58)
LVEF1 TE	61.75 (SD 3.85)
LVEF2 TE	59.54 (SD 4.12)

**Table 6:** Left ventricular ejection fraction comparison before and after both antibody drug treatments using *t*-test

Ejection fraction comparison	*t*-test of paired samples (mean ± SD), %
LVEF1 T versus LVEF2 T	64.69 ± 4.84 versus 60.58 ± 6.58 *t* = 4.96, df = 25, *p* < 0.001
LVEF1 TE versus LVEF2 TE	61.75 ± 3.85 versus 59.54 ± 4.12 *t* = 7.65, df = 23, *p* < 0.001
LVEF1 T versus LVEF 1 TE	64.69 ± 4.84 versus 61.75 ± 3.85 *t* = 2.2, df = 23, *p* = 0.038
LVEF1 T versus LVEF 2 TE	64.69 ± 4.84 versus 59.54 ± 4.12 *t* = 3.7, df = 23, *p* = 0.001
LVEF2 T versus LVEF 1 TE	60.58 ± 6.58 versus 61.75 ± 3.85 *t* = 1.08, df = 23, *p* = 0.29
LVEF2 T versus LVEF2 TE	60.58 ± 6.58 versus 59.54 ± 4.12 *t* = 0.36, df = 23, *p* = 0.716

We used Student’s *t*-test of paired samples to compare LVEF before and after trastuzumab and trastuzumab emtansine protocols

## Conclusion

In conclusion, trastuzumab and trastuzumab emtansine had no effect on PQ and QRS interval duration, but have shown a significant effect on QTc interval duration. There was a significant QTc interval prolongation, already seen with first application of both drugs, with further significant QTc prolongation after each successive drug cycle, reaching the peak value before the fifth cycle. Effects of both drugs on QTc interval prolongation were reversible, and posttreatment values returned to approximately starting levels in both protocols. Our results also confirm clinically silent but statistically significant LVEF reduction after both drug protocols, with a partial LVEF recovery after the trastuzumab treatment. There was no correlation between QTc interval duration and LVEF, measured before or after treatment with both drug protocols (Additional files 1, 2 and 3).

## Supplementary Information

**Additional file 1. Table 1.** The table shows the values of PQ, QRS and QTc intervals before each cycle (PQ1-6, QRS1-6, QTc1-6) as well as the values of PQ7, QRS7 and QTc7 after the last cycle of trastuzumab for each individual patient. Average heart rate values for each individual patient as well as the age of each patient at the time of trastuzumab therapy are also shown.

**Additional file 2. Table 1.** The table shows the values of PQ, QRS and QTc intervals before each cycle (PQ1-6, QRS1-6, QTc1-6) as well as the values of PQ7, QRS7 and QTc7 after the last cycle of trastuzumab emtansine for each individual patient. Average heart rate values for each individual patient as well as the age of each patient at the time of trastuzumab emtansine therapy are also shown.

**Additional file 3. Table 1.** The table shows the values of ejection systolic function for each patient before (LVEF1) and after (LVEF2) administration of transtuzumab and before and after administration of trastuzumab emtansine (LVEF1, LVEF2).

## Data Availability

The datasets supporting the conclusions of this article are included within the article (and its additional file).

## Abbreviations

QTc: Corrected QT interval
LVEF: Left ventricular ejection fraction
HER2+: Human epidermal growth factor receptor 2 positive
ECG: Electrocardiogram
EGFR: Epidermal growth factor receptor
T-DM1: Trastuzumab emtansine
*T*: Test statistic
Df: Degrees of freedom
*P*: Statistical significance
SD: Standard deviation
*R*: Rho coefficient
BF10: Bayesian factor 10
CMP: Cardiomyopathy
SCD: Sudden cardiac death
TdP: Torsade de pointes
EAD: Early after-depolarization
VPC: Ventricular premature contraction
